# Recurrent respiratory infections and vitamin A levels: a link? It is cross-sectional

**DOI:** 10.1097/MD.0000000000030108

**Published:** 2022-08-19

**Authors:** Ashraf Abdelkader, Ashraf A. Wahba, Mohamed El-tonsy, Amr Abdelfattah Zewail, Mohamed Shams Eldin

**Affiliations:** a Department of Pediatrics, Faculty of Medicine (Boys), Al-Azhar University, Cairo, Egypt; b Scientific Research and Continuous Medical Education Unit, Al Ansari Specialist Hospital, Yanbu, Saudi Arabia; c Department of Otorhinolaryngology, Faculty of Medicine, Al-Azhar University, Damietta, Egypt; d Department of Clinical Pathology, Faculty of Medicine, Al-Azhar University, Assiut, Egypt; e Department of Basic Science, Vision Medical College, Jeddah, Saudi Arabia; f Department of Otorhinolaryngology, Faculty of Medicine, Al-Azhar University, Cairo, Egypt

**Keywords:** Infection, Recurrent, Respiratory, Vitamin A

## Abstract

Respiratory tract infections are common illnesses in children, causing significant morbidity and negatively affecting their health. Vitamin A protects against infections and maintains epithelial integrity. The goal of this study was to determine the correlation between vitamin A deficiency and recurrent respiratory tract infections (RRTIs). Participants in this cross-sectional study were divided into 3 groups: RRTIs (including patients with history of RRTIs presenting with respiratory tract infection symptoms), RTI (including patients without history of RRTIs presenting with respiratory tract infection symptoms), and control (including children who came for a routine health checkup without a history of RRTIs or respiratory tract infection symptoms). The vitamin A assay was performed using high-performance liquid chromatography. The study included 550 children aged 6.64 ± 2.61 years. The RRTIs group included 150 children (27.3%), the RTI group included 300 children (54.5%), and the control group included 100 children (18.2%). Subclinical vitamin A deficiency and vitamin A deficiency affected 3.1% and 1.3% of subjects, respectively. Subclinical vitamin A deficiency and vitamin A deficiency were higher in children with RRTIs than in those with RTI (8% vs 1.3%, *P* = .001 and 4% vs 0.3%, *P* = .006). Additionally, children with RRTIs had significantly higher rates of subclinical vitamin A deficiency and vitamin A deficiency than those in the control group, which had 1% subclinical vitamin A deficiency (*P* = .017) and no cases of vitamin A deficiency (*P* = .043). The RRTIs group had higher rates of otitis media (27.3%), sinusitis (20%), and pneumonia (4.7%) than the RTI group (*P* = .002). Vitamin A insufficiency was associated with RRTIs in children.

## 1. Introduction

Vitamin A is water-insoluble, but fat-soluble.^[1]^ Retinol, retinal, and retinoic acid (RA) are all forms of vitamin A, with RA having the most biological action. The principal biological activities include maintaining vision, development, and epithelial and mucous tissue integrity.^[2]^ It is well known that retinoic acid is important in the regulation of differentiation, maturation, and function of cells of the innate immune system.^[3,4]^

Respiratory tract infections are the most frequent infectious illnesses in children and adolescents and are a major public health issue that causes significant morbidity and mortality, putting children’s health at risk. Preschool-aged children who have had more than 8 bouts of airway infections each year or older children who have had more than 6 respiratory infections in the absence of any underlying pathological conditions are considered to have recurrent respiratory tract infections (RRTIs).^[5]^ In children with RRTIs aged 0 to 2, 3 to 5, and 6 to 14 years, infectious episodes occur more than 7, 6, and 5 times per year, respectively.^[6]^

Any upper or lower respiratory sickness, as well as any respiratory ailment accompanied by fever (axillary temperature greater than or equal to 37.5°C or rectal temperature greater than or equal to 38°C), is considered a respiratory tract infection.^[7]^ At least one of the following symptoms is usually present: runny nose, nasal congestion, sore throat, cough, earache, wheezing, and/or shortness of breath lasting for at least 2–3 days. Recurrent episodes should be separated by at least a 2-week period during which no symptoms are experienced.^[7]^

Traditional RRTIs risk factors include parental education level, family medical history of respiratory diseases, parental smoking status, nutritional status, supplemental microelements, immune status, dietary status, exercise, and environmental factors.^[8]^

Previous studies have suggested that low serum vitamin A levels are associated with high RRTIs and respiratory infection morbidity.^[9,10]^ In accordance with these findings, it has been discovered that patients with chronic infectious diseases are more likely than the general population to present with severe vitamin A deficiency.^[11]^ An additional study was conducted on 200 children where 63.5% of them received vitamin A supplementation, while 36.5 did not, and found that the number of acute lower respiratory tract infections (ALRTIs) in children who got vitamin A supplementation was significantly reduced (<3 episodes per year).^[12]^ As a result, this study was designed to investigate if there was a link between blood vitamin A levels and the occurrence of RRTIs in children, as well as to assess how severe respiratory infection symptoms were in vitamin A deficient children.

## 2. Patient and Methods

This cross-sectional study was performed between April 2019 and May 2021. The participants of the current study were selected from children who visited the pediatric or ENT clinics at Al Ansari Specialist Hospital in Yanbu, Saudi Arabia, for the treatment of respiratory tract symptoms or a regular physical examination and routine checkup. The study protocol was approved by the Institutional Scientific Research Ethics Committee (No.19-4/5).

The participants and their families were informed before the study, and the parents of the children provided written informed permission for research enrollment and data access.

The patients were divided into 3 groups: the RRTIs group (including patients who presented with respiratory tract symptoms and had a history of RRTIs), the RTI group (including patients who presented with respiratory tract symptoms without a history of RRTIs), and the control group (including children who came for a routine health checkup without a history of RRTIs or any respiratory symptoms).

Respiratory tract infection was defined as any upper or lower respiratory symptoms accompanied by fever (axillary temperature greater than or equal to 37.5°C or rectal temperature greater than or equal to 38°C) for at least 2–3 days.^[5]^

All patients in the RRTIs group met the definition of recurrent respiratory tract infection, which is defined as the occurrence of 8 or more documented airway infections per year in children under 3 years of age, or 6 or more in children older than 3 years of age, with recurrent episodes separated by at least a 2-week symptom-free interval in the absence of any underlying pathological condition.^[5,13]^

Children under the age of 18 months were excluded from this study. In addition, all children with a medical condition affecting the respiratory tract, such as bronchial asthma, patients with a history of parental smoking or chronic exposure to air pollution, those with a history of immune deficiency, those taking any medication that had an effect on their immune status, such as corticosteroids or immunosuppressive agents, those who had acute or chronic inflammation of other systems or organs, congenital diseases, or a history of recent major surgery, which included (duration of surgery more than 90 minutes, intraperitoneal or extensive extraperitoneal dissection, or a postoperative stay of more than 24 hours),^[14]^ were excluded from this study.

All the children underwent a standard medical examination, which included measurements of height, weight, and temperature, as well as a detailed clinical examination of their upper respiratory tract and chest. Body mass index (BMI) was calculated by dividing the body weight (kg) by the height in meters squared (m^2^). BMI age and sex-specific percentile curves (for 2–20 years) were then used to determine nutritional status as follows: underweight children’s BMI percentiles were lower than the 5th percentile, overweight children’s BMI percentiles ranged from the 85th to 95th percentile, and BMI percentiles of obese children exceeded the 95th percentile.^[15]^

After overnight fasting, 3 mL of peripheral venous blood was drawn into thrombin blood collection tubes (orange top) for each participant. Blood samples were collected and centrifuged for 10 minutes at 3000 rpm/min before being allowed to settle. The supernatant was then transferred to a clean microcentrifuge tube. The serum was collected and stored at 20°C until testing. Our hospital laboratory employed High-performance liquid chromatography (HPLC) was used to evaluate the serum vitamin A concentrations (Cobas e 411-Japan). Vitamin A levels > 0.3, <0.3, and <0.2 mg/L were classified as normal, subclinical deficiency, and deficiency, respectively, according to the World Health Organization deficiency standards.^[16]^

All procedures detailed here were performed by a professional pediatrician and otolaryngologist, including history taking and physical examination.

### 2.1. Statistical analysis

The analysis was performed using SPSS software package (version 25.0; IBM SPSS Statistics for Windows (Armonk, NY: IBM Corp.). For descriptive statistics, mean ± SD was used for quantitative variables, while frequency and percentage were used as qualitative variables. Fisher exact test was used to assess differences in the frequency of qualitative variables. The correlation between continuous variables was assessed using Pearson correlation coefficient. The statistical methods were verified; all tests were 2-sidedassuming a significant level of *P* < .05 and a highly significant level of *P* < .001 with 80% power and a 95% confidence interval.

## 3. Results

Among the 550 children who met the inclusion and exclusion criteria, with a mean age of 6.64 ± 2.61 years, and ages ranging from 2 to 12 years, approximately 58.5% were males. The RRTIs group included 150 patients (27.3%), RTI group included 300 patients (54.5%), and control group included 100 healthy children (18.2%). Of the participants, 74.5% were of normal weight, 8.9% were underweight, and around 16.6% were overweight or obese, with an overall mean BMI of 20.5 ± 4.23, which ranged from 11.5 to 35 kg/m^2^. In both respiratory groups, 66% reported colds and pharyngitis, 21.6% reported otitis media, 10.6% reported sinusitis, and 1.8% reported being sick with pneumonia. Vitamin A levels averaged 0.33 ± 0.17 mg/L and ranged from 0.14 to 0.84 mg/L. Approximately 3.1% and 1.3% of participants had subclinical vitamin A deficiency and vitamin A deficiency, respectively (Table 1).

**Table 1:** General and clinical characteristics of the studied sample.

Variables		n = 550 (%)
Age (years)	Mean ± SD	6.64 ± 2.61
	Min—Max	2–12
	Preschool (2 – <6)	316 (57.5)
	School (6–12)	234 (42.5)
Gender	Male	322 (58.5)
	Female	228 (41.5)
BMI (kg/m^2^)	Mean ± SD	20.5 ± 4.23
	Min—max	11.5–35
Nutritional status	Underweight	49 (8.9)
	Normal	410 (74.5)
	Overweight/obese	91 (16.6)
Respiratory condition	RRTIs	150 (27.3)
	RTI	300 (54.5)
	Normal	100 (18.2)
Respiratory illness (n = 450)	Cold	156 (34.7)
	Pharyngitis	141 (31.3)
	Otitis media	97 (21.6)
	Sinusitis	48 (10.6)
	Pneumonia	8 (1.8)
Vitamin A (mg/L)	Mean ± SD	0.33 ± 0.17
	Min—max	0.14–0.84
	Normal	526 (95.6)
	Subclinical deficiency	17 (3.1)
	Deficiency	7 (1.3)

RRTIs: recurrent respiratory tract infections; RTI: respiratory tract infection.

The participants in each of the 3 groups were similar in term of age and sex. Subclinical vitamin A deficiency and vitamin A deficiency were significantly higher in children with RRTIs than in those with RTI (8% vs 1.3%, *P* = .001 and 4% vs 0.3%, *P* = .006, respectively). Additionally, children with RRTIs had significantly higher rates of vitamin A subclinical deficiency and vitamin A deficiency than those in the control group, which had 1% subclinical vitamin A deficiency (*P* = .017) and no cases of vitamin A deficiency (*P* = .043). Underweight was significantly more prevalent among children with RRTIs than in the RTI (16.7% vs 7%, *P* = .003) or control groups (16.7% vs 3%, *P* = .001). Compared with the RTI group, the RRTIs group was more frequently diagnosed with otitis media (27.3% vs 18.7%, *P* = .039), sinusitis (20% vs 6%, *P* = .001), and pneumonia (4.7% vs 0.3%, *P* = .002) (Table 2).

**Table 2:** Characteristics of the studied sample according to respiratory condition.

Variables	RRTIs n = 150 (%)	RTI n = 300 (%)	Control n = 100 (%)	*P* *	*P* †	*P* ‡
Age (years)						
Preschool (2–<6)	76 (50.7)	179 (59.7)	61 (61.0)	0.086	0.121	0.906
School (6–12)	74 (49.3)	121 (40.3)	39 (39.0)			
Gender						
Male	90 (60.0)	177 (59.0)	55 (55.0)	0.919	0.436	0.485
Female	60 (40.0)	123 (41.0)	45 (45.0)			
Nutritional status						
Underweight	25 (16.7)	21 (7.0)	3 (3.0)	0.003§	0.001§	0.222
Normal	96 (64.0)	234 (78.0)	80 (80.0)	0.002§	0.007§	0.779
Overweight/obese	29 (19.3)	45 (15.0)	17 (17.0)	0.281	0.739	0.634
Respiratory illness						
Cold	28 (18.7)	128 (42.7)	–	<0.001§	–	–
Pharyngitis	44 (29.3)	97 (32.3)	–	0.590	–	–
Otitis media	41 (27.3)	56 (18.7)	–	0.039§	–	–
Sinusitis	30 (20.0)	18 (6.0)	–	<0.001§	–	–
Pneumonia	7 (4.7)	1 (0.3)	–	0.002§	–	–
Vitamin A levels						
Normal	132 (88.0)	295 (98.3)	99 (99.0)	<0.001§	0.001§	1.000
Subclinical deficiency	12 (8.0)	4 (1.3)	1 (1.0)	0.001§	0.017§	1.000
Deficiency	6 (4.0)	1 (0.3)	0 (0.0)	0.006§	0.043§	1.000

* Difference between RRTIs and RTI groups.

† Difference between RRTIs and control groups.

‡ Difference between RTI and control groups.

§ Significant.

Vitamin A levels did not differ significantly by age or sex among the 3 groups. Underweight was significantly more common in the vitamin A deficiency and subclinical deficiency groups than in the group with appropriate vitamin A levels (42.9% vs 7.6%, *P* = .014 and 35.3% vs 7.6%, *P* = .002, respectively). In terms of respiratory diseases, both deficiency groups (subclinical vitamin A deficiency and vitamin A deficiency) had a higher rate of pneumonia (29.4% and 28.6%, respectively, *P* < .001 each) than normal-level children (0.2%). Similarly, the subclinical deficiency group had a significantly higher rate of sinusitis (35.3%, *P* = .002) than the normal-level group (7.6%) (Table 3).

**Table 3:** Characteristics of the studied sample according to the serum vitamin A levels.

Variables	Normal* n = 526 (%)	Subclinical vitamin A deficiency n = 17 (%)	Vitamin A Deficiency n = 7 (%)	*P* †	*P* ‡	*P* §
Age (years)						
Preschool (2 – < 6)	300 (57.0)	12 (70.6)	4 (57.1)	0.325	1.000	0.647
School (6–12)	226 (43.0)	5 (29.4)	3 (42.9)			
Gender						
Male	307 (58.4)	12 (70.6)	3 (42.9)	0.454	0.459	0.356
Female	219 (41.6)	5 (29.4)	4 (57.1)			
Nutritional status						
Underweight	40 (7.6)	6 (35.3)	3 (42.9)	0.002∥	0.014∥	1.000
Normal	398 (75.7)	9 (52.9)	3 (42.9)	0.045∥	0.067	1.000
Overweight/obese	88 (16.7)	2 (11.8)	1 (14.2)	0.751	1.000	1.000
Respiratory illness						
Cold	155 (29.5)	1 (5.9)	0 (0.0)	0.031∥	0.202	1.000
Pharyngitis	140 (26.6)	1 (5.9)	0 (0.0)	0.087	0.198	1.000
Otitis media	90 (17.1)	4 (23.5)	3 (42.9)	0.513	0.106	0.374
Sinusitis	40 (7.6)	6 (35.3)	2 (28.6)	0.002∥	0.099	1.000
Pneumonia	1 (0.2)	5 (29.4)	2 (28.6)	<0.001∥	<0.001∥	1.000

* Normal level vitamin A group includes 100 children with no respiratory symptoms.

† Difference between Normal and Subclinical deficiency groups.

‡ Difference between Normal and Deficiency groups.

§ Difference between Subclinical deficiency and Deficiency groups.

∥ Significant.

A significant positive correlation was found between BMI and serum vitamin A levels (a decrease in BMI was associated with a decrease in vitamin A levels (*R* = 0.147, *P* = .001) (Table 4).

**Table 4:** Correlation between BMI and serum vitamin A levels.

		Vitamin A level
BMI	r	0.147
	*P*-value	0.001*

r: Pearson correlation coefficient.

* Significant.

## 4. Discussion

Respiratory tract infections are caused by pathogenic microorganisms, and can be classified into 2 types. Upper and lower respiratory tract infections. Upper respiratory tract infections (rhinitis, pharyngitis, otitis media, sinusitis, and laryngitis) are diagnosed at a higher rate than lower respiratory tract infections (tracheitis, bronchitis, bronchiolitis, and pneumonia).^[17,18]^ Children with weakened immunity are more likely to develop respiratory tract infections, which can progress to recurrent respiratory tract infections (RRTIs).^[19]^

Consistent results were ensured by the lack of a statistically significant difference in age and sex across groups, as well as the exclusion of any disorder that could impact the health of the respiratory tract.

Our findings indicate that RRTIs are more prevalent in children with subclinical vitamin A deficiency, vitamin A deficiency, and underweight than in the general population. Additionally, this study found that patients with RRTIs are more likely to develop more serious respiratory illnesses, such as otitis media, sinusitis, or pneumonia, than those with nonrecurrent respiratory diseases.

Although the current study identified no link between vitamin A deficiency and age or sex, vitamin A was commonly associated with children with nutritional deficiency, which may be a component of overall vitamin and other element paucity due to the decline in retinol binding protein production.^[20]^

The current study is consistent with prior randomized controlled trials and double-blind studies revealing that vitamin A supplementation can boost the immunity of patients with chronic vitamin A deficiency.^[21,22]^ Zhang et al discovered a correlation between low serum vitamin A levels and recurrent respiratory tract infections (P 0.05).^[10]^ According to Murthy et al reported that children who received vitamin A supplementation had a significantly lower incidence of recurrent acute lower respiratory tract infections than those who did not receive vitamin A supplementation (P 0.001).^[12]^

A significant association between vitamin A deficiency and RRTI, as well as the severity of disease development observed by this study, can be explained by the fact that micronutrient deficiencies, such as a deficiency of vitamins and minerals, are associated with a variety of medical conditions, including an increased risk of developing upper and lower RTIs.^[23]^ Vitamin A deficiency alters the respiratory epithelium and impairs humoral as well as cellular immunity.^[24]^ Vitamin A appears to be critical for mammalian body growth and immune system development. Common infectious diseases such as measles, diarrhea, and respiratory tract infections are caused by a weakened immune response.^[25]^ Immunoglobulin A (IgA), a key indicator of the mucosal immune system, has previously been linked to respiratory infection.^[26]^ In previous studies, Vitamin A has been shown to be critical for T-cell differentiation, IgA secretion, and class switching.^[27–29]^ Additionally, vitamin A deficiency can affect mucoprotein expression and lymphocyte proliferation in response to antigen activation, reducing the immune response in patients’ airway mucus,^[30]^ which is composed of mucoproteins and glycoproteins that form a protective barrier against antigens.^[31]^ Taken together, these findings indicate that vitamin A deficiency can impair immune function and increase the risk of infection.

This study is limited by its cross-sectional design. It was not possible to establish a causal association between low serum vitamin A levels and RRTIs. Furthermore, we were unable to distinguish between vitamin A insufficiency and RRTIs in terms of time sequence; therefore, we recommend investigating the effect of vitamin A supplementation in a population with RRTI. Our findings should also be interpreted with caution because they only apply to children in Yanbu, Saudi Arabia, and a larger native-wide study is needed to determine the link between vitamin A and recurrent respiratory infections in children in KSA. Finally, possibility of residual confounding caused by unmeasured covariates (such as the possible effect of socioeconomic status) can’t be excluded.

## 5. Conclusion

Vitamin A deficiency, which is more prevalent in malnourished children, was found to be associated not only with an increased incidence of RRTIs, but also with the severity of RTI symptoms in children. Nutritional assessment and correction, as well as vitamin A supplementation, may be effective in reducing RTI symptoms and RRTIs in children.

## Acknowledgments

In particular, we would like to express our heartfelt appreciation and gratitude to Dr Alaa Abdelqader Altaweel, professor of Maxillofacial, Faculty of Dentist Al-Azhar University for Boys, for his never-ending enthusiasm and support throughout the study process, even after the project came close.

## Author contributions

Abdelkader A designed and wrote the manuscript; Abdelkader A, Shams Eldin M, Wahba A, and El-tonsy M collected data and provided patients’ clinical information; Zewail A analyzed the data. All the authors have read and approved the final manuscript.
